# Association between inflammation‐related indicators and vertebral fracture in older adults in the United States: A cross‐sectional study based on National Health and Nutrition Examination Survey 2013–2014

**DOI:** 10.1002/iid3.70047

**Published:** 2024-11-07

**Authors:** Yuwei Gou, Xiansong Xie, Heng Yin, Yucheng Wu, Yongjie Wen, Yingbo Zhang

**Affiliations:** ^1^ Department of Orthopedics Affiliated Hospital of North Sichuan Medical College Nanchong Sichuan China

**Keywords:** inflammation‐related index, NHANES, vertebral fracture

## Abstract

**Objective:**

This study was to examine the association between inflammation‐related indexes SII (systemic immune‐inflammation index), NLR (neutrophil‐to‐lymphocyte ratio), PPN (product of platelet count and neutrophil count), and PLR (platelet‐to‐lymphocyte ratio), and the occurrence of vertebral fractures in older Americans.

**Methods:**

Patients ⩾60 years of age from the 2013–2014 NHANES database were selected for this study. Restricted cubic spline models and weighted logistic regression models were used to assess the association between inflammation‐related indexes and the occurrence of vertebral fractures in older Americans. The predictive value of the inflammation‐related indexes on the occurrence of vertebral fractures was assessed using receiver operating characteristic curves (ROC). To examine the robustness of the main findings, a sensitivity analysis was conducted.

**Results:**

A total of 1281 patients were included in the analysis, of whole 120 suffered vertebral fractures. Fully adjusted logistic regression showed a significant linear relationship between NLR and the occurrence of vertebral fracture in older Americans (*p* < .05), but no relationship was found between SII, PLR, and the occurrence of vertebral fracture in older Americans. Meanwhile, NLR was slightly better than other indicators in predicting vertebral fracture.

**Conclusions:**

This study found that NLR, as a novel inflammatory marker, can predict the risk of vertebral fracture in older Americans, which is of clinical significance for the prevention and treatment of vertebral fracture in older adults.

## 1 INTRODUCTION

Vertebral fracture is a common type of fracture in older adults and is considered to be closely related to osteoporosis.^1^ Besides, the high incidence of vertebral fracture is closely related to the decline of patients' survival rate.^1^ Most studies have found that at least one‐fifth of patients over 50 years old have one or more spinal fractures.^2^ Studies have shown that the incidence of low‐energy trauma in patients aged 60 and above is four times that of young people, the incidence of thoracolumbar fractures is 30 per 100,000 people every year, and about two‐thirds of patients are over 60 years old.^3^ Vertebral fractures are more common in postmenopausal women, which can lead to acute and chronic pain, dysfunction, and even disability, and decline in quality of life. Kyphosis may occur in the long run, which can last for several years, increase mortality, and decrease life expectancy.^4^ Nevertheless, there is no consensus on the best treatment for vertebral fractures. Therefore, it is of great significance to implement appropriate intervention for vertebral fractures as soon as possible to reduce their risk, improve the quality of life, and extend lifespan.

Inflammation‐related index is a comprehensive indicator integrating the counts of platelets, neutrophils, and lymphocytes, and has been proven to be a promising prognostic indicator for many diseases, including malignant tumors, coronary artery diseases, acute ischemic stroke, premature rupture of membranes, infectious diseases, and autoimmune diseases.^5^, ^6^, ^7^, ^8^ It has been demonstrated that SII may be used to predict the occurrence of brittle fractures in older patients with osteoporosis, and systemic inflammatory reactions are involved in fractures and acute inflammatory responses after trauma in the preoperative phase in older adults.^9^ Previous studies have found that a high level of SII (≥834.89) is relatively accurate in predicting osteoporotic fractures in postmenopausal women.^8^, ^10^ Therefore, evaluating the risk of vertebral fracture in older adults based on the novel index of blood cell count may be beneficial to preventing vertebral fracture in older adults.

Currently, there is no research to investigate the relationship between inflammation‐related index and vertebral fracture. Therefore, the purpose of this study is to explore the relationship of systemic immune‐inflammation index (SII), platelet‐to‐lymphocyte ratio (PLR), neutrophil‐to‐lymphocyte ratio (NLR), and the product of platelet count and neutrophil count (PPN) with vertebral fracture.

## 2 METHODS

### 2.1 Samples and data sources

Relevant samples and data used in this study were all derived from National Health and Nutrition Examination Survey (NHANES) between 2013 and 2014. NHANES is a continuous cross‐sectional research survey managed by the National Center for Health Statistics (NCHS) to evaluate the nutritional and health status of adults and children in the United States. This study was approved by the Research Ethics Review Committee of the National Center for Health Statistics and obtained the informed consent from the participants.

### 2.2 Study design and population

**Figure 1:**
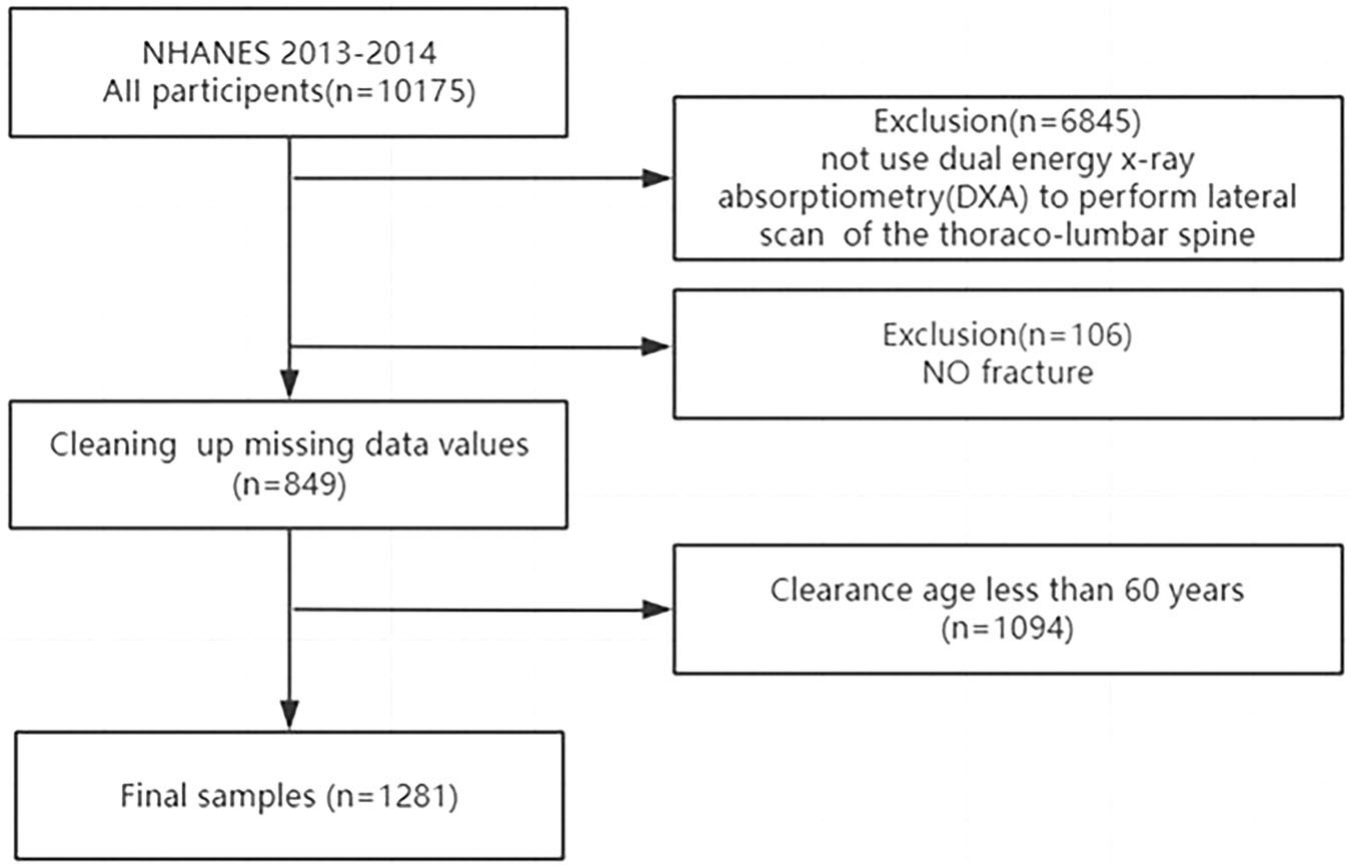
Detailed patient recruitment flowchart.

In the NHANES 2013–2014, among all 10,175 subjects, 6845 patients who did not receive dual‐energy X‐ray, 106 patients who did not diagnosed with vertebral fracture after receiving dual‐energy X‐ray, 849 patients with missing covariate and inflammatory index data and 1094 patients under 60 years old were excluded. Finally, 1281 patients were included in the analysis. The process is presented in Figure 1.

Inclusion criteria as follows: Patients were included if they were ≥60 years old in NHANES from 2013 to 2014. Exclusion criteria were as follows: Patients were excluded if (1) they were <60 years old; (2) they were not evaluated by dual‐energy X‐ray absorptiometry; (3) there was a lack of SII, NLR, or PLR data; (4) the covariate data were missing. In this study, the Mobile Examination Center (MEC) weights were applied.

### 2.3 Definition and measurement of vertebral fracture

The vertebral fracture was measured by the lateral view of the thoracolumbar spine with dual‐energy X‐ray absorptiometry (DXA). The image resolution of the lateral spine scan obtained by DXA is close to that of a standard radiograph. The accuracy of DXA VFA and standard radiograph in detecting moderate to severe vertebral fractures is similar (defined by Genant's semi‐quantitative criteria (1993).^11^ Lateral DXA scanning of the thoracolumbar spine was performed in the NHANES MEC from 2013 to 2014. According to the Vertebral Fracture Status Summary, the vertebral status was given 1 for normal (no fracture between T4‐L4, unevaluable vertebra in T7‐L4 ≤ 1), 2 for fracture (mild, moderate, or severe fracture between T4‐L4), and 3 for unexplained status (no fractures and unevaluable vertebra between T7‐L4 ≤ 1). In this study, any level of mild, moderate, or severe fractures between T4‐L4 were defined as vertebral fractures.

### 2.4 Measurement of inflammatory indicators

SII, NLR, PPN, and PLR were used as inflammatory indicators in this study. Beckman Coulter DxH 800 in the NHANES MEC was utilized to count blood samples and provide blood cell distribution of all participants. The Beckman Coulter method of counting and sizing was used to derive complete blood count (CBC) parameters, combined with automatic dilution, and mixing equipment for sample processing and a single‐beam spectrophotometer for hemoglobin determination. The VCS technique was applied in differential counting of whole blood cell count (WBC). The following formula was used for calculation: SII = (platelet count × neutrophil count)/lymphocyte count, PLR = platelet count/lymphocyte count, NLR = neutrophil count/lymphocyte count; PPN = platelet count × neutrophil count.

### 2.5 Covariate selection

Considering the influence of other factors on vertebral fracture, this study included factors that may affect the risk of vertebral fractures, such as age, gender (male or female), smoking history (smokers or nonsmokers; smokers were defined as having smoked at least 100 cigarettes in their lifetime), drinking history (drinker or nondrinker: drinker was defined as drinking at least 12 cups of alcoholic beverages a year, including beer, wine and any other type of alcoholic beverages. “At least 12 cups of any kind of alcoholic beverage” was defined as 12 ounces of beer, 5 ounces of wine, or 1.5 ounces of liquor), hypertension (with or without hypertension; hypertension was defined as ever being told of having hypertension by doctors), diabetes (with or without diabetes; diabetes was defined as ever being told of having diabetes by doctors), height, weight, work activities (intense work activities or nonintense work activities: intense work activities were defined as work involving intense activities that lead to a sharp increase in breathing or heart rate, such as carrying or lifting heavy objects, digging or building for at least 10 min), osteoporosis (with or without osteoporosis; osteoporosis was defined as ever being told of having osteoporosis), vitamin D, and race (Mexican American, other Hispanics, Non‐Hispanic whites, Non‐Hispanic Blacks, and other races—including multiraces).

### 2.6 Statistical analysis

NHANES adopted a complex, multistage, probability sampling design. Shapiro–Wilk test was adopted to evaluate data distribution. For data of normal distribution, continuous variables were expressed as mean ± standard deviation, and data of non‐normal distribution were expressed as median and interquartile range in brackets. *χ* ^2^, Mann–Whitney *U* test or independent *t* test were conducted to compare the differences between the two groups. In this study, a weighted univariate logistic regression was used to screen the influencing factors of vertebral fracture risk in older adults in the United States. Weighted multivariate logistic regression (WLMR) models were applied to evaluate the association of SII, NLR, PPN, and PLR with vertebral fracture in older adults in the United States. The rough model was initially fitted without adjustment, and then the influencing factors with *p* < .05 were incorporated into subsequent models for adjustment. The results were expressed by the ratio (OR) and its 95% confidence interval (CI). The area under the curve (AUC) was calculated to evaluate the predictive ability of SII, NLR, PPN, and PLR for the risk of vertebral fracture in older adults in the United States. The restricted cubic spline (RCS) model was used to study the linear or nonlinear relationship of SII, NLR, PPN, and PLR with vertebral fracture in older adults in the United States. To examine the robustness of the main findings, a two‐step sensitivity analysis was conducted: in Model 1, an unweighted logistic regression model was adopted; in Model 2, a weighted logistic regression after excluding patients diagnosed with osteoporosis at baseline was conducted. The statistical software Rstudio 4.3.1 was used, and a *p* value threshold of .05 or less was considered statistically significant (bilateral).

## 3 RESULTS

### 3.1 Baseline characteristics of participants

A total of 1281 patients were included in this study, including 615 males (46%) and 666 females (54%), with an average age of 68 years. There was a statistical difference between the two groups in age, and participants in the fracture group were older than those of the nonfracture group (*p* < .001). We found that there was statistical significance between osteoporosis, diabetes, and vertebral fracture in older adults in America (*p* < .05). There was a statistical difference in weight between the two groups (*p *< .05). Baseline characteristics of participants are provided in Table 1.

**Table 1:** Baseline information on inflammation and fracture.

Characteristic	*N* 1	Overall, *N* = 46,001,7002	0, *N* = 41,926,346^2^	1, *N* = 4,075,354^2^	*p* Value^3^
Drink	1281	873 (73%)	789 (74%)	84 (70%)	.5
Hypertension	1281	810 (60%)	730 (60%)	80 (60%)	>.9
Gender	1281				.7
Female		666 (54%)	602 (54%)	64 (57%)	
Man		615 (46%)	559 (46%)	56 (43%)	
Age	1281	68 (63,74)	68 (63,74)	74 (65,80)	.001
Race	1281				.3
Mexican American		141 (4.3%)	131 (4.3%)	10 (3.7%)	
Non‐Hispanic Black		257 (8.4%)	243 (8.6%)	14 (6.3%)	
Non‐Hispanic White		648 (79%)	564 (79%)	84 (85%)	
Other Hispanic		105 (3.0%)	100 (3.1%)	5 (1.7%)	
Other race—Including multiracial		130 (5.2%)	123 (5.4%)	7 (3.3%)	
Diabetes	1281	313 (21%)	290 (21%)	23 (14%)	.019
0steoporosis	1281	176 (14%)	148 (13%)	28 (24%)	.014
Physical activity	1281	147 (15%)	136 (15%)	11 (9.1%)	.3
Smoke	1281	643 (50%)	580 (49%)	63 (56%)	.3
VitamineD	1281	80 (63,98)	80 (63,97)	76 (61,107)	>.9
Stature	1281	66 (63,70)	66 (63,70)	65 (62,68)	.074
Weight	1281	172 (148,198)	173 (148,200)	162 (140,186)	.02
SII	1281	465 (338,665)	458 (336,652)	555 (359,710)	.057
PLR	1281	118 (95,149)	119 (95,146)	115 (95,155)	.8
NLR	1281	2.14 (1.61,2.94)	2.11 (1.59,2.89)	2.44 (1.71,3.50)	.014
PPN	1281	54 (42,69)	54 (43,70)	49 (37,68)	.056

^1^ *N* not missing (unweighted).

^2^ *n* (unweighted) (%); median (25%,75%).

^3^ *χ* ^2^ test with Rao and Scott's second‐order correction; Wilcoxon rank‐sum test for complex survey samples.

### 3.2 Association between covariates and vertebral fracture

The univariate logistic regression analysis showed that age, weight, osteoporosis, and diabetes were the risk factors of vertebral fracture in older individuals in America (*p *< .05). They were included as confounders in the weighted multivariate logistic regression analysis. as shown in Table 2.

**Table 2:** Association between covariates and vertebral fracture.

	Factor	Level	OR	CI	*p* Value
1	Drink	NO	Ref		
	Drink	YES	0.84	0.49–1.45	.53
2	Hypertension	NO	Ref		
	Hypertension	YES	0.98	0.59–1.63	.95
3	Gender	Female	Ref		
	Gender	Male	0.89	0.48–1.63	.71
4	Age		1.09	1.04–1.14	0
5	Race	Non‐Hispanic Black	0.84	0.35–1.99	.7
	Race	Non‐Hispanic White	1.25	0.56–2.78	.59
	Race	Other Hispanic	0.65	0.14–2.95	.59
	Race	Other race—Including multiracial	0.71	0.17–2.94	.64
6	Diabetes	NO	Ref		
	Diabetes	YES	0.58	0.38–0.87	.02
7	0steoporosis	NO			
	0steoporosis	YES	2.18	1.25–3.81	.02
8	Physical activity	NO	Ref		
	Physical activity	YES	0.56	0.21–1.48	.26
9	Smoke	NO	Ref		
	Smoke	YES	1.3	0.8–2.09	.31
10	Vitamin D		1	1–1.01	.51
11	Stature		0.93	0.88–0.99	.05
12	Weight		0.99	0.99–1	0

### 3.3 Association between SII, PLR, NLR and PPN, and vertebral fracture

Weighted multivariate logistic regression was used to analyze the association between SII, NLR, PPN, and PLR and vertebral fracture in older adults. Model 1, in which confounding factors were not adjusted, showed that vertebral fractures were statistically significantly associated with SII, PPN, and NLR (*p *< .05) in older adults in the United States, but not significantly correlated with PLR. After adjusting the factors of age, osteoporosis, weight and diabetes in Model 2, the results of weighted multivariate logistic regression showed that there was a significant positive correlation between NLR and the risk of vertebral fracture in older adults in the United States (OR: 1.192, 95% CI: 1.035–1.374), but the relationship between SII, PLR and PPN and the occurrence of vertebral fractures in older adults in the United States was not significant, as illustrated in Table 3.

**Table 3:** Association of SII, PLR, NLR, PPN with vertebral fracture.

		Model 1			Model 2	
	OR	95% CI	*p* Value	OR	95% CI	*p* Value
SII	1.001	1.000, 1.001	.017	1.001	1.000, 1.001	.051
PLR	1.002	0.996, 1.007	.5	1.001	0.995, 1.007	.7
NLR	1.211	1.071, 1.369	.005	1.192	1.035, 1.374	.019
PPN	0.987	0.976, 0.998	.023	0.987	0.976, 0.999	.037

*Note*: Model 1: Unadjusted; Model 2: Adjusted for age, osteoporosis, weight, and diabetes.

Abbreviations: NLR, neutrophil‐to‐lymphocyte ratio; PLR, platelet‐to‐lymphocyte ratio.

### 3.4 Predictive ability of different inflammatory markers on the risk of vertebral fracture in older adults in the United States

**Figure 2:**
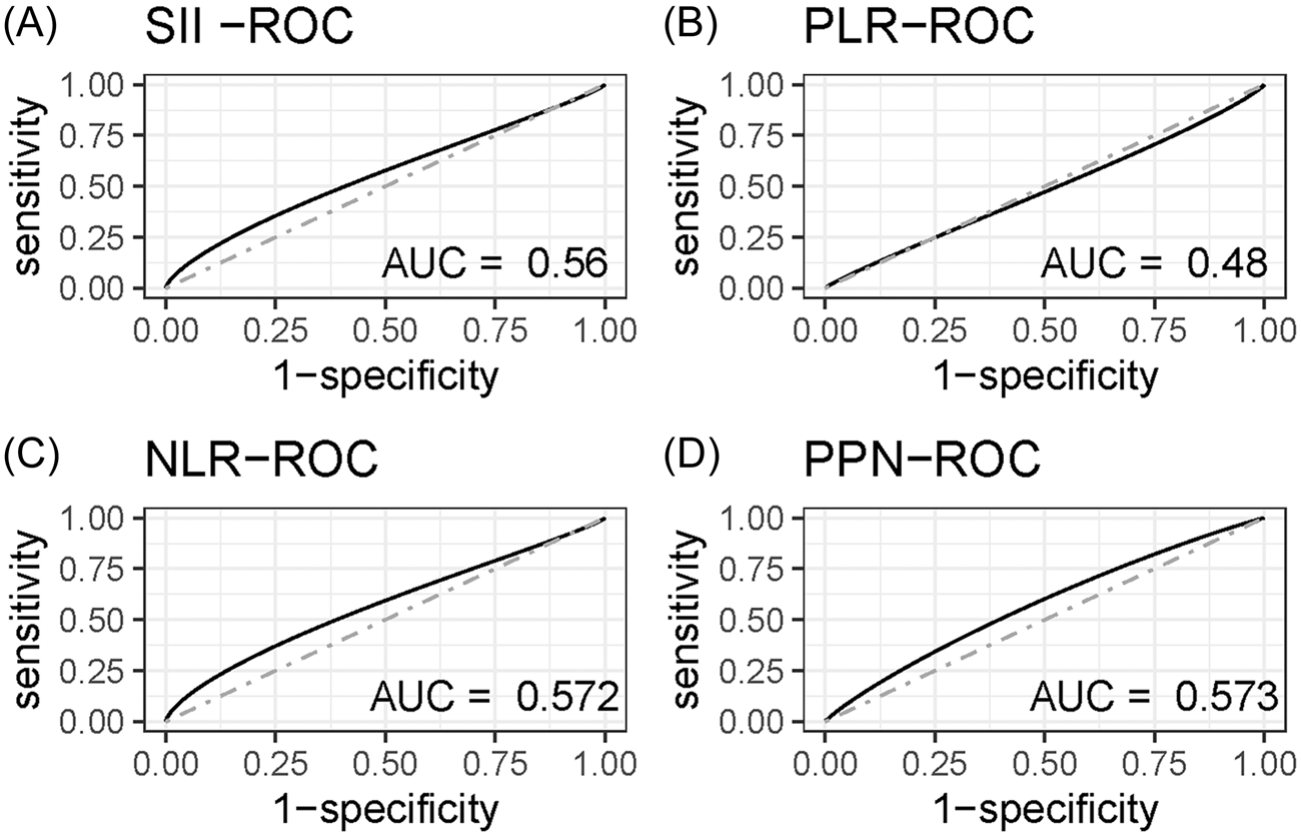
Predictive ability of different inflammatory markers on the risk of vertebral fracture in elderly in the United States. (A) SII. (B) PLR. (C) NLR. (D) PPN. NLR, neutrophil‐to‐lymphocyte ratio; PLR, platelet‐to‐lymphocyte ratio.

The receiver operating characteristic (ROC) curve was used to compare the performance of different inflammatory markers for predicting the risk of vertebral fracture in older adults in the United States. The results revealed that compared with PLR (AUC = 0.48) and SII (AUC = 0.56), NLR (AUC= = 0.572), and PPN (AUC = 0.573) were more effective in predicting the risk of vertebral fracture in older adults in the United States (Figure 2).

### 3.5 Linear or nonlinear relationship between SII and vertebral fracture in older adults using restricted cubic spline (RCS) model

**Figure 3:**
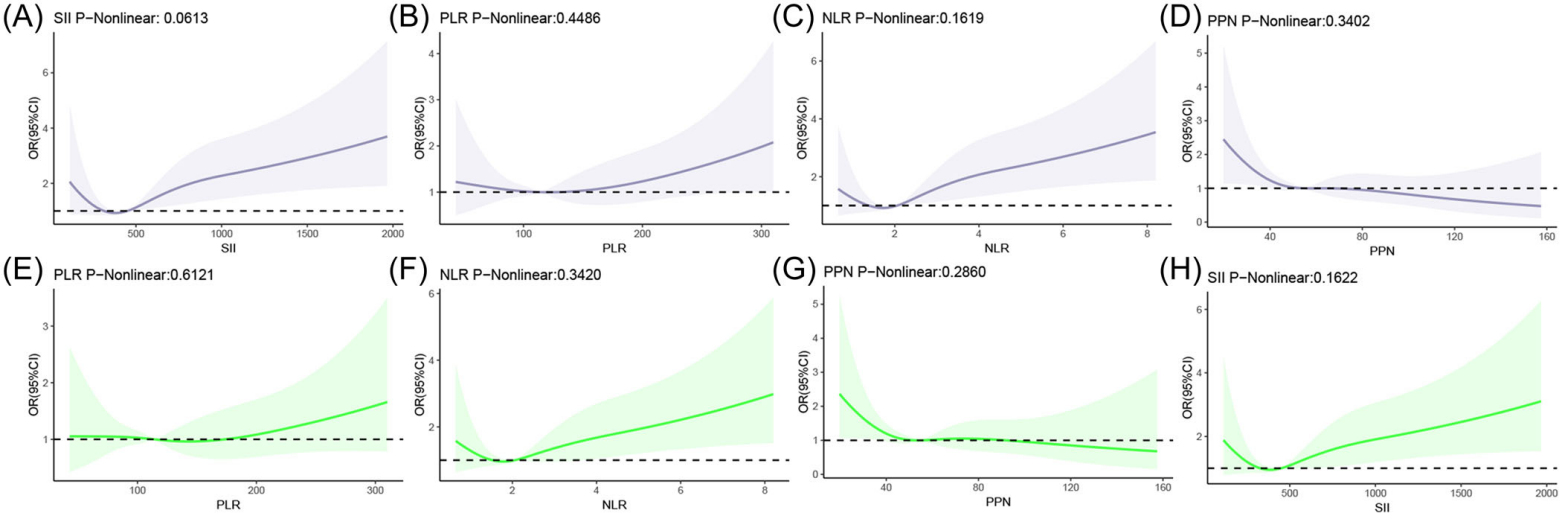
(A–D) Unadjusted restricted cubic spline (RCS) model; (E–H) RCS model adjusted for confounding factors.

The linear and nonlinear relationship was assessed between SII, NLR, PPN and PLR, and vertebral fracture in older adults. A U‐shaped linear relationship was observed between SII and the risk of vertebral fracture in older adults (*p* nonlinear > .05), and there was a linear relationship between NLR, PPN and PLR, and the risk of vertebral fracture in older adults. When SII > 500, a continuous increase in SII value was linked with a significantly elevated risk of vertebral fracture in older adults. After adjusting for factors of age, osteoporosis, weight and diabetes, a linear relationship was observed between SII, NLR, PPN, and PLR and vertebral fracture, as depicted in Figure 3.

### 3.6 Sensitivity analyses

To examine the robustness of the main findings, a sensitivity analysis was conducted. First, the unweighted multiple logistic regression Model 1 showed that there was still a statistically significant association between NLR and vertebral fractures in older Americans (Model 1: OR: 1.139, 95% CI: 1.028–1.265, *p *< .05). Second, after excluding patients diagnosed with osteoporosis at baseline, the weighted logistic regression Model 2 also showed a consistent association between NLR and the risk of vertebral fractures in older Americans (Model 2: OR: 1.187, 95% CI: 1.037–1.393, *p *< .05) (Table 4).

**Table 4:** Sensitivity analysis.

		Model 1			Model 2	
	OR	95% CI	*p* Value	OR	95% CI	*p* Value
NLR	1.139	1.028, 1.265	.013	1.187	1.037, 1.393	.026

*Note*: Model 1: Using unweighted logistic regression models; Model 2: Weighted logistic regression model for excluding patients diagnosed with osteoporosis at baseline, (adjusted for age, osteoporosis, weight, and diabetes).

## 4 DISCUSSION

In this study, the association between inflammatory markers (SII, NLR, PPN, and PLR) and the risk of vertebral fracture in older adults in the United States was investigated. Among these markers, NLR was positively correlated with the occurrence of vertebral fractures in older adults in the United States, while SII, PPN, and PLR were not significantly correlated with the risk of vertebral fractures. The results also showed that SII, NLR, PPN, and PLR had a linear relationship with vertebral fracture after adjusting confounders. In addition, NLR had a better predictive ability for the risk of vertebral fracture in older adults.

Previous studies have shown that osteoporosis and weakened bone strength are important causes of vertebral fracture.^12^, ^13^ This study found that compared with the group without osteoporosis, the risk of vertebral fracture in the osteoporosis group increased significantly. Osteoporosis can lead to a decrease in bone mass and an increase in bone brittleness, further leading to vertebral fracture without obvious inducement and external force.^14^ Meanwhile, there are many reasons for vertebral fractures. In this study, age was found to be a contributor to vertebral fractures. Wright et al.^15^ reveal that the incidence of hip and spine fractures in women depends on age. BMI is reported as an important risk factor for osteoporosis,^16^ which is related to vertebral fractures, and weight is also related to vertebral fractures. In this study, osteoporosis, age, and weight were related to the occurrence of vertebral fractures in older adults, so these confounding factors were further adjusted.

Similar to SII, other novel indicators of chronic inflammation, such as NLR, PLR, and PPN, can also reflect the level of systemic immune inflammation by integrating the relationship between various leukocyte subtypes. Ma et al.^17^ have found that there are many kinds of inflammatory cells in the bone marrow cavity, and the cascade reaction of inflammatory cells is related to the dysfunction of lymphocytes, which leads to the aggregation of neutrophils and macrophages, further resulting in the breaking of the dynamic balance of bone, the inhibition of bone formation and the induction of bone resorption. Hajishenglis et al.^18^ found that higher levels of neutrophils can cause more inflammatory factors to induce bone resorption. Studies have shown that inflammatory cytokines (CRP, IL‐1) act on mesenchymal stem cells and osteoclasts to enhance osteoclast‐mediated bone resorption.^19^ Studies have also confirmed that bone remodeling is related to chronic inflammation, which may be related to oxidative stress and immune activation.^20^ Previous studies have found that with the increase of age, the immune system will be in a subclinical inflammatory state, which can affect the function of lymphocytes and lead to an imbalance between inflammatory and anti‐inflammatory factors in bone metabolism.^21^ It was reported that some inflammatory factors (IL‐1, IL‐9, and others) can promote osteoclast differentiation or inhibit osteogenic differentiation of bone marrow mesenchymal stem cells by activating the RANKL signaling pathway.^22^ Lee et al.^23^ also found that in chronic inflammation, neutrophils can release reactive oxygen species through RANKL signaling and increase osteoclast differentiation to promote osteoblast apoptosis. Barbour et al.^19^ also found that chronic inflammatory factors (NLR, PLR) can bind to stromal cells to activate RANKL signaling and reduce the production of osteoprotegerin, thus elevating the activity of osteoclasts. Hofbauer et al.^24^ held that osteoprotegerin (OPG) and RANKL signaling are related to bone loss. Bord et al.^25^ have proved that PIT plays an important role in bone formation, resorption and homeostasis. Also, some researchers attach importance to the effect of PIT on bone formation, and they think platelet‐derived growth factor (PDGF) can promote extracellular matrix and promote the formation of bone.^26^ Therefore, during osteopenia, PIT increases while lymphocytes decrease, and PLR and the risk of osteoporotic fracture increase, which is consistent with our research results. Studies by Herrero‐Cervera et al.^27^ and Koupenova et al.^28^ showed that neutrophils and PIT can be activated in an inflammatory state, which can be expressed by NLR and PLR values. The increase of inflammatory cytokines can affect osteoclasts through various ways, and even bone resorption, indicating that high levels of NLR and PLR will lead to metabolic bone diseases and osteoporotic fractures.^29^, ^30^ Hajishenglis et al.^18^ have found that neutrophils can secrete CCL2 and CCL20 chemokines to recruit Th17 cells, and activated neutrophils that migrate to bones can express RANKL, enhance the activity of osteoclasts and promote the occurrence of fractures. SII and NLR have been proven to be related to the state of systemic inflammatory reaction.^31^ The cascade reaction of inflammatory cells and chemokines in the inflammatory reaction is triggered by lymphocytes, which leads to the aggregation of neutrophils and macrophages, affects the balance of bone metabolism, and weakens bone strength, leading to the occurrence of fractures.^32^ The results of this study also showed that inflammatory markers are associated with the risk of vertebral fracture in older adults in the United States, and higher levels of SII and NLR are positively correlated with the risk of vertebral fracture in older adults.

Some previous studies have explored the relationship between inflammatory indicators and osteoporosis. Tang et al.^8^ have found that SII can better predict the risk of low bone mineral density (BMD) or osteoporosis in postmenopausal women, indicating that osteoporosis is closely related to high levels of SII, NLR and PPN. Zhang et al.^33^ have found that the level of SII in the osteoporosis group is significantly higher than that in the nonosteoporosis group, and the results proved that there is a correlation between osteoporosis and higher SII. The research of Fang et al.^10^ also found that higher SII can increase the risk of postmenopausal osteoporosis, which is an important predictor of the diagnosis of postmenopausal osteoporosis. In a study by Huang et al.^34^ it was found that the increase in NLR level is related to osteopenia in postmenopausal women. Also, Liu et al.^35^ proved that a high level of NLR is related to osteopenia. Fang et al.^10^ observed in 238 postmenopausal women in China that an SII level of ≥834.89 is a risk factor for osteoporosis. A study by Qu et al.^36^ proved that NLR is significantly related to osteoporosis or osteopenia, and indicated that SII and NLR are related to the mortality of patients with osteopenia. A cross‐sectional study found that high levels of NLR and PLR are closely related to the prevalence of osteoporosis, which proved that NLR and PLR can be used as effective indicators for screening osteoporosis.^37^ Yilmaz et al.^38^ reported that the level of NLR in the osteoporosis group was significantly higher than that in the bone health group, and they emphasized that NLR can be used as a reliable predictor in postmenopausal osteoporosis women. This study also found that a higher level of NLR was related to the occurrence of vertebral fracture in older adults, which is consistent with those research results. In addition, some previous studies have investigated the relationship between osteoporosis, BMD and fracture, BMD and inflammatory indicators, which found that osteoporosis can affect the deterioration of bone structure, lead to osteopenia, and increase the risk of fracture and the incidence of related complications.^39^, ^40^ Tang et al.^8^ observed that the increase of NLR and PPN levels is related to the decrease of BMD and the increase of osteoporosis risk, and also found that PPN is negatively correlated with BMD in any bone site, which suggested that PPN is an inflammatory index for the decrease of BMD. In a study on the correlation between inflammatory markers and BMD, Chen et al.^41^ found that NLR and PLR are associated with lumbar spine BMD, indicating that PLR can be used as a potential inflammatory predictor of osteoporosis and is superior to NLR. Koseoglu^42^ proved in a clinical study that NLR iss associated with lumbar spine BMD. Their results showed that in postmenopausal women, PLR level can predict low BMD through baseline measurement, and there is a close correlation between PLR and bone loss. The results of this study also showed that NLR is related to the occurrence of vertebral fractures in older adults, and its prediction efficiency is better than PLR, SII, and PPN.

Our research has demonstrated the correlation between NLR and vertebral fractures. Its ability to distinguish between the presence and absence of vertebral fractures is limited. However, the research results still provide valuable significance. Some studies have found that elevated levels of NLR are associated with osteoporosis in postmenopausal women and have demonstrated that NLR can serve as an effective indicator for screening osteoporosis.^36^, ^39^ As an easily accessible and cost‐effective inflammatory marker, the association between NLR and vertebral fractures may suggest its value as an auxiliary diagnostic tool. Although its discrimination performance is limited, it has value as an auxiliary diagnostic tool. It is suggested that future research should explore whether NLR, in specific situations or in combination with other inflammatory markers, can help improve the ability to identify high‐risk patients.

Studies have shown that inflammation is closely related to brittle fractures.^43^ Some studies have reported the correlation between inflammatory biomarkers (SII, PLR, NLR) and fracture.^40^, ^44^ Huang et al.^34^ have found in a study that NLR is an independent risk factor leading to osteoporotic vertebral fracture, and can predict the risk of such fracture, which is similar to our research results. Our research indicated that there was a correlation between inflammatory markers (SII, PLR, NLR) and vertebral fractures in the old populations, and suggested the value of NLR as an auxiliary diagnostic tool and its ability to determine the risk of vertebral fractures in specific old populations. In addition, the sensitivity analysis revealed a certain correlation between NLR and vertebral fractures in patients without osteoporosis. In general, as an independent risk factor for vertebral fractures, NLR has certain value in predicting vertebral fractures.

To sum up, this study revealed that inflammatory markers have an impact on the risk of vertebral fracture in older individuals in the United States, but there are some limitations in this cross‐sectional study. A cross‐sectional study only collects data at one time point and can only display associations among variables, but cannot confirm whether one variable causes changes in another variable. Due to the simultaneous collection of data, this study lacks a temporal dimension and cannot reveal the changes in variables over time or whether the observed changes have sustainability. Multiple confounders were identified through questionnaires. Using questionnaires to obtain a large sample coverage population may result in low response rates, which may also affect the interpretation of the results. Therefore, it is relatively difficult to explore causal relationships through cross‐sectional studies.^45^ To validate the findings of this study and further explore the relationship between inflammatory factors and vertebral fractures, it is necessary to increase and implement longitudinal studies in the future to track changes in the same population over time, so as to better understand the impact of inflammatory factors on the risk of vertebral fractures.

In addition, although we have adjusted for some confounders, there may still be some potential confounders, such as physical activity level and dietary factors. Research has shown that as the main source of bioactive compounds, diet may mediate inflammatory responses,^46^ and studies have also found that pro‐inflammatory diets are associated with fractures in the older adults.^47^ Studies have shown that physical activity is beneficial for bone health, and appropriate weight‐bearing exercises can increase muscle mass and bone mineral density.^48^ There are also studies indicating that the basal level of inflammation does not affect the exercise‐induced changes in skeletal outcomes in relatively healthy middle‐aged and older populations.^49^ To better control for potential confounders, more comprehensive variables should be collected in the future studies to ensure appropriate adjustments for all factors that may affect the study results, to better understand the relationship between inflammatory markers and vertebral fractures.

## 5 CONCLUSIONS

This study shows that NLR is related to the occurrence of vertebral fractures in older adults in the United States. As a novel inflammatory marker, it can predict the risk of vertebral fractures in older adults in the United States, and exhibits better performance than SII, PLR, and PPN.

## AUTHOR CONTRIBUTIONS

**Yuwei Gou**: Writing—original draft preparation; writing—review and editing; conceptualization; methodology; formal analysis and investigation. **Xiansong Xie**: Writing—review and editing. **Yucheng Wu**: Conceptualization. **Heng Yin**: Conceptualization. **Yongjie Wen**: Methodology. **Yingbo Zhang**: Formal analysis and investigation; funding acquisition; resources; supervision. All authors commented on previous versions of the manuscript. All authors read and approved the final manuscript. All authors contributed to the study conception and design.

## CONFLICT OF INTEREST STATEMENT

The authors declare no conflict of interest.

## ETHICS STATEMENT

This study was based on a public database and approved by the Research Ethics Review Committee of the National Center for Health Statistics (NCHS). All participants provided written informed consent.

## Supporting information

Supporting information.

## Data Availability

The original contributions presented in the study are included in the article, further inquiries can be directed to the corresponding author.
